# Mesenchymal Stem Cells and Myeloid Derived Suppressor Cells: Common Traits in Immune Regulation

**DOI:** 10.1155/2016/7121580

**Published:** 2016-07-27

**Authors:** Irina Lyadova Vladimirovna, Ekaterina Sosunova, Alexander Nikolaev, Tatiana Nenasheva

**Affiliations:** Central Tuberculosis Research Institute, Yauza Alley 2, Moscow 107564, Russia

## Abstract

To protect host against immune-mediated damage, immune responses are tightly regulated. The regulation of immune responses is mediated by various populations of mature immune cells, such as T regulatory cells and B regulatory cells, but also by immature cells of different origins. In this review, we discuss regulatory properties and mechanisms whereby two distinct populations of immature cells, mesenchymal stem cells, and myeloid derived suppressor cells mediate immune regulation, focusing on their similarities, discrepancies, and potential clinical applications.

## 1. Introduction

Immune response protects host against pathogen invasion and cancer. However, if uncontrolled, it may induce severe tissue damage and therefore under steady-state conditions is tightly regulated. Understanding cells and mechanisms that regulate immune response is critical to unravel pathogenesis of many diseases and develop new strategies for immune modulation during cancer, chronic infections, autoimmune disorders, allergies, and following organ transplantation.

Several populations of immune cells have been implicated in the control of immune response including natural and induced CD4^+^ T regulatory cells (Treg), CD8^+^ Treg, Breg, macrophages, and dendritic cells. To control immune response, these cells utilize a set of core suppressive mechanisms, the main of which are the secretion of inhibitory cytokines (e.g., IL-10, TGF-*β*, and IL-35), the expression of inhibitory receptors (e.g., PD-L1), the inhibition of antigen-presenting cell maturation, and cytolysis [1–4].

Besides mature immunocompetent cells designated to control immune response, other populations may also contribute to immune regulation. In particular, two distinct populations of functionally immature cells, mesenchymal stem cells (MSCs), and a population of immature myeloid cells, myeloid derived suppressor cells (MDSCs), have been implicated in immune suppression and regulation [5, 6].

MSCs and MDSCs belong to distinct differentiation lineages; however, their immunoregulatory properties have several common traits. Here, we review the underlying mechanisms and regulatory properties of MSCs and MDSCs focusing on their similarities and distinctions.

## 2. MSCs and MDSCs: General Characteristics

### 2.1. MSCs

MSCs are multipotent stromal self-renewing cells capable to differentiate into mesenchymal tissues like osteocytes, chondrocytes, and adipocytes [7]. MSCs exhibit paracrine effects and participate in immunomodulation and tissue repair. The cells are found in the bone marrow (BM) and other embryonic and adult tissues such as cord blood, placenta, adipose tissue, and perivascular sources. In the BM, MSCs fulfill a supportive function for hematopoietic cells and participate in the control of their renewal and differentiation [8–10]. Phenotypically, MSCs are characterized by the expression of CD105, CD90, and CD73 and lack of the expression of haemopoietic markers, such as CD45, CD34, CD14, CD11b, CD79*α*, CD19, and HLA-DR [11–13].

The immunomodulatory properties of MSCs were first demonstrated by Di Nicola and coauthors, who showed that BM-MSCs inhibited T cell proliferation in mixed lymphocyte reaction (MLR) [14]. Since then, the ability of MSCs to suppress immune responses has been extensively studied. Currently, it is understood that MSCs possess rather immunoregulatory than immunosuppressive properties: depending on the microenvironment they can inhibit, modulate or even enhance immune function of various immune cells [5, 15]. Proinflammatory conditions induce suppressive properties in MSCs. Due to their immunoregulatory properties and the feasibility of generating the large numbers of autologous MSCs, MSCs are considered as a potentially valuable tool for clinical immunomodulation.

### 2.2. MDSCs

MDSCs belong to the hematopoietic lineage and represent the heterogeneous population of early myeloid progenitors/precursors of granulocytes, macrophages, and dendritic cells (DCs) able to mediate immune suppression [6]. In steady-state conditions, MDSCs are rare and are primarily found in the BM. During different pathologies accompanied by inflammation, MDSCs accumulate abundantly in the BM, blood, spleen, lungs, and other organs [16–19].

In mice, MDSCs are defined as Gr-1^+/dim^CD11b^+^ cells. In human, MDSCs are generally identified based on the expression of CD33 and CD11b and lack of the expression of HLA-DR. Two main subsets of MDSCs, monocytic and granulocytic, have been described according to their nuclear morphology and phenotype. In mice, monocytic and granulocytic MDSCs are identified as Ly-6G^−/low^Ly-6C^hi^CD11b^+^ (F4/80^+^CD115^+^CD49d^+^) and Ly-6G^+^Ly-6C^low^CD11b^+^ (F4-80^−^CD115^−^CD49d^−^) cells, respectively. In human, they are CD14^+^HLA-DR^−^ and CD15^+^/CD66b^+^, respectively [6, 19–22].

MDSCs are the main negative regulators of immune response in cancer [6, 23] and are also involved in the immune suppression in many other pathological conditions [19, 24–26]. Similar to MSCs, the suppressive activity of MDSCs is inducible and dramatically increases under proinflammatory conditions [6]. Expressed immunosuppressive properties and abundant accumulation of MDSCs under proinflammatory conditions make them an attractive target for immunomodulation in cancer and other diseases.

## 3. Molecular Mediators of Immune Suppression

To regulate immune response, MSCs and MDSCs utilize a set of mediators and mechanisms, which they partially share with other immune regulatory cells. In this section, we summarize the main mechanisms whereby MSCs and MDSCs mediate immune regulation focusing on their cellular targets and activation.

### 3.1. Indoleamine 2,3-dioxygenase (IDO) and Tryptophan Metabolism

#### 3.1.1. Effects

**Figure 1:**
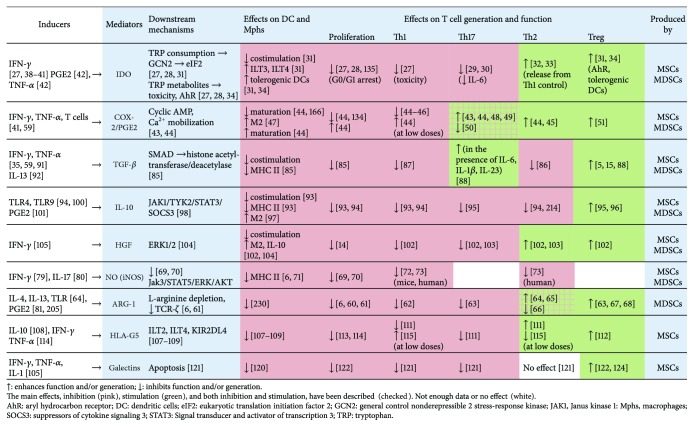
Main mediators utilized by MSCs and MDSCs to suppress T cell responses and their effects.

IDO enzymes (expressed as two distinct enzymes, IDO1 and IDO2) catalyze the essential amino acid tryptophan into metabolites, that is, kynurenine, quinolinic acid, and picolinic acid. Tryptophan consumption increases the level of uncharged tryptophan tRNA in immune cells. This activates general control nonderepressible 2 (GCN2) stress-response kinase, eukaryotic translation initiation factor 2 (eIF2), and GCN2-eIF2-mediated pathway, which leads to the reduction in protein synthesis, retards cellular proliferation, arrests T cells in G0/G1 cell cycle, and increases lymphocyte sensitivity to Fas-mediated apoptosis [27, 28]. The activation of GCN2-mediated pathway also downregulates IL-6 supporting the suppressive status of Tregs and restricting their conversion to Th17-like cells [29]. In a model of Th17-associated experimental autoimmune encephalomyelitis (EAE), halofuginone, a small molecule that induces amino acid starvation, selectively inhibited the differentiation of Th17, verifying a role of amino acid deficiency in the suppression of Th17 [30]. Tryptophan depletion decreases the expression of costimulatory molecules and increases the expression of the inhibitory receptors ILT3 and ILT4 by DCs; DCs differentiated under low-tryptophan conditions become tolerogenic [31] (Figure 1).

Tryptophan metabolites are directly toxic to CD8^+^ and CD4^+^ Th1 cells, whereas Th2 cells are more resistant to their toxicity. Therefore, IDO releases Th2 from Th1-mediated suppression and skews T helper response towards Th2 type [27, 32, 33]. Kynurenines are also natural ligands for the aryl hydrocarbon receptor (AhR); their interaction with AhR promotes the differentiation of CD4^+^Foxp3^+^ Treg cells, interferes with the generation of Th17, and decreases the immunogenicity of DCs [34].

#### 3.1.2. IDO Expression by MSCs and MDSCs

In the immune system, IDO are expressed primarily by professional antigen-presenting cells [28]. Both MSCs and MDSCs express IDO and utilize IDO mediated mechanisms for immune suppression [35–37].

#### 3.1.3. Regulation of IDO Expression

The IDO gene is inducible in the presence of IFN-*γ* and regulated by upstream IFN-*γ*-responsive elements that bind activated STAT1, interferon regulatory factor-1 (IRF-1), and NF-*κ*B [27, 35, 38, 39]. IRF-8 contributes to IFN-*γ*-induced IDO expression by enhancing the expression of IDO and decreasing DAP12 which basally opposes IDO expression [40]. It was suggested that MSCs utilize IDO mediated mechanism in the presence of IFN-*γ* but not in basal state [41]. IDO expression is also increased by PGE2 [42], thus relating the two mechanisms of immune control utilized by MSCs and MDSCs.

### 3.2. Cyclooxygenase-2 and Prostaglandin E2

#### 3.2.1. Effects

PGE2 synthesizes from the arachidonic acid after the latter releases from membrane phospholipids and is metabolized by either the constitutive cyclooxygenase-1 (COX-1) or the inducible cyclooxygenase-2 (COX-2) [43]. PGE2 mediates pain, edema, and fever, the main features of inflammation. At the same time, it exerts anti-inflammatory effects. The interaction of PGE2 with EP2 and EP4 receptors expressed by immune cells leads to increase in cyclic AMP, activates protein kinase A and phosphatidylinositol-3 kinase dependent signaling pathways, and inhibits Ca^2+^ mobilization. Cyclic AMP interferes with IL-2-mediated pathways, inhibits the expression of proinflammatory cytokines and chemokines (i.e., IL-12p70, TNF-*α*, CCL3, and CCL4), and induces the expression of IL-10, IL-4, and IL-5 [43, 44]. This suppresses cell proliferation, induces alternative macrophages (M2), stimulates Th2, and weakens Th1 responses [44–47]. Besides inducing immunosuppression, PGE2 may play proinflammatory role in T cell function. In some studies, exogenous PGE2 enhanced DC maturation and T cell proliferation (reviewed in [44]). The above-mentioned inhibition of IL-12p70 induced by PGE2 is due to the inhibition of IL-12p35 chain. IL-12p40 chain is not affected by PGE2. IL-12p40 and p19 form IL-23, the cytokine involved in the generation of Th17. PGE2 increases the expression of p19 resulting in the increased production of IL-23 and Th17 polarization [43, 48]. In a model of EAE, EP4 antagonist decreased the accumulation of Th17 and Th1 cells and suppressed disease progression [49]. However, in some other studies, PGE2 inhibited Th17 differentiation [50]. Recent advances suggest that effects of PGE2 on Th17 and even Th1 depend on its concentration in such a way that micromolar concentrations suppress the responses while nanomolar concentrations promote the responses [44].

PGE2 enhances the differentiation of Foxp3^+^ Treg cells [51], elevates TGF-*β* secretion by monocytes, and induces the generation of MDSCs and their accumulation in tumor environment. The inhibition of COX-2 suppresses these processes [52–54].

#### 3.2.2. Regulation of COX-2/PGE2 and Their Expression by MSCs and MDSCs

Both MSCs and MDSCs express COX-2 [41] and can produce PGE2 [41, 54–58]. PGE2 production increases in inflammatory conditions, that is, in the presence of IFN-*γ* and TNF-*α* and after cell coculture with peripheral blood cells [41, 59].

### 3.3. Arginase-1, Inducible Nitric Oxide Synthase, and Arginine Metabolism

#### 3.3.1. Effects

Arginase-1 (ARG1) hydrolyses L-arginine to ornithine and urea reducing local arginine concentration. The latter activates GCN2, which inhibits cell cycling [60]. ARG1 downregulates the *ζ*-chain of the T cell receptor (TCR) complex, disturbing the process of T cell activation [6, 61]. There is only limited data on the subset-specific effects of ARG1. The studies have reported the inhibition of IFN-*γ* [62] and Th17 [63], and both the stimulation [64, 65] and the suppression [66] of Th2 responses by ARG1 produced by various cells. Tregs are expanded by ARG1; the inhibitor of ARG1 N-hydroxy-L-arginine (NOHA) abrogates this effect [67, 68].

Besides ARG1, L-arginine is metabolized by inducible nitric oxide (NO) synthase (iNOS) that generates NO. NO suppresses T cell function through the inhibition of JAK3, STAT5, ERK, and AKT involved in IL-2 signaling and the control of T cell proliferation [69, 70]. NO also inhibits the expression of MHC class II and induces T cell apoptosis [6, 71]. In murine T cells, NO was shown to suppress the secretion of Th1 cytokines [72]; in human T cells, it suppressed the secretion of both Th1 and Th2 cytokines [73].

#### 3.3.2. ARG1 and iNOS Expression by MSCs and MDSCs

In the immune system, ARG1 and iNOS are generally expressed by polymorphonuclear cells (PMN) and monocyte/macrophages [74]; T helper cells are also able to produce NO [72]. In M1 and M2 macrophages, ARG1 and iNOS are expressed reciprocally: ARG1 is expressed by M2, whereas iNOS by M1 subset [75]. MDSCs express both ARG1 and iNOS [6, 70]; however, the levels of their expression in monocytic and granulocytic populations may differ so that ARG1 is expressed predominantly by granulocytic MDSCs [76] and iNOS by monocytic MDSCs [6]. MSCs express iNOS and can produce NO [77], but there is no evidence for their expression of ARG1. In spite of this, MSCs can contribute to the depletion of L-arginine by promoting the generation of MDSCs [78].

#### 3.3.3. The Regulation of ARG1 and iNOS

Generally, ARG1 and iNOS undergo reciprocal induction: ARG1 is induced by Th2 cytokines, whereas iNOS by Th1 cytokines [79]. Recently, IL-17 was shown to contribute to iNOS expression by enhancing its mRNA stability [80]. PGE2 stimulates ARG1 [81].

### 3.4. Reactive Oxygen Species and Peroxynitrite

#### 3.4.1. Effects

Reactive oxygen species (ROS) are generated by NADPH oxidase which produces superoxide anion (O_2_^−^). Superoxide anion reacts with NO to form peroxynitrite. Peroxynitrate oxidates membrane molecules and nitrates amino acids. Nitration of TCRs alters antigen-recognition and inhibits the responses of CD4^+^ and CD8^+^ cells [82]. Nitration of the chemokine CCL2 was shown to block T cell migration to the inflammatory site [83].

#### 3.4.2. ROS Production by MSCs and MDSCs

NADPH oxidase is generally expressed by leukocytes. In MDSCs, it is expressed predominantly by the granulocytic population [6]. MSCs do not generate ROS, but they are responsive to them: ROS promote MSCs' aging. In physiological levels, ROS improve MSCs' proliferation and differentiation [84].

### 3.5. Cytokines and Growth Factors

The main immunoregulatory cytokines produced by MSCs and MDSCs are TGF-*β* and IL-10.

#### 3.5.1. TGF-*β*

TGF-*β* binds to the heterodimeric TGF-*β* receptor and initiates SMAD-dependent and SMAD-independent signal transduction pathways. SMAD-dependent pathway induces the recruitment of histone acetyltransferase and deacetylase to the promoters of target genes [85]. This leads to the blockade of IL-2 production, downregulates cell cycle promoting factors, upregulates cyclin-dependent kinase inhibitors, and inhibits the expression of MHC class II and costimulatory molecules in DCs and effector molecules (i.e., IFN-*γ* and perforin) in T cells. Consequently, the proliferation, helper, and cytotoxic activity of T and NK cells are suppressed. TGF-*β* inhibits the differentiation of both Th1 and Th2 cells [86, 87]. In contrast, it promotes the generation of Treg and Breg cells. TGF-*β* is a key regulator of Foxp3 expression [5, 15, 88]. In the presence of IL-6, IL-1*β* or IL-23 TGF-*β* promotes the generation of Th17 [88]. Recently, TGF-*β* was shown to inhibit the expression of iNOS in MSCs reversing their suppressive effect on T cell proliferation and manifesting immunostimulatory effect [89].

MSCs constitutively secrete TGF-*β* [90] and upregulate its production in the inflammatory environment, that is, in the presence of IFN-*γ* and TNF-*α* [35, 59, 91]. In MDSCs, the expression of TGF-*β* was induced by IL-13 [92].

#### 3.5.2. IL-10

IL-10 is produced by various immune cells, including DCs, macrophages, Th1, Th2, Th17, Treg, CD8^+^ T cells, and B lymphocytes, and also by MSCs and MDSCs. IL-10 directly acts on antigen-presenting cells (APC), decreasing their maturation, and the expression of MHC and costimulatory molecules [93]. IL-10 inhibits the production of proinflammatory cytokines and chemokines (i.e., IL-1*α*, IL-1*β*, IL-6, IL-12, TNF-*α*, CCL2, and CCL5, IL-8) and hampers DC migration to lymph nodes and the generation of effector T cells. Direct effects of IL-10 on T lymphocytes include the inhibition of proliferation, IL-2, IFN-*γ*, TNF-*α*, IL-4, and IL-5 production and memory formation [93, 94]. IL-10 also inhibits the differentiation of Th17 [95] but enhances the differentiation of IL-10 producing Treg cells and M2 macrophages [96, 97]. The anti-inflammatory effects of IL-10 are mediated through the phosphorylation of JAK1, TYK2, the activation of STAT3, and the induction of SOCS3, which negatively regulates various cytokine genes [98]. Apart from the immunosuppressive activity, IL-10 may display immunostimulatory properties: it inhibited or stimulated CD8^+^ T cells depending on the type of pathogen and cell microenvironment [99]. Induction of IL-10 goes on as a result of toll-like receptors (TLR4 and others) activation [100]. PGE2 is a potent inducer of IL-10 [101].

#### 3.5.3. Hepatocyte Growth Factor (HGF)

HGF is primarily produced by MSCs. HGF displays pleiotropic immunosuppressive activity. It stimulates IL-10 production by monocytes, downregulates costimulatory molecules on DCs, inhibits Th1, and induces IL-10 producing Treg cells [102–105]. HGF produced by MSCs expanded human MDSCs [78]. One study reported the production of HGF by tumor-infiltrating MDSCs [106], which highlights the similarities between the two populations.

MSCs and MDSCs produce a number of other cytokines that contribute to cell biological activity, for example, IL-6, IL-8, and GM-CSF.

### 3.6. Other Mechanisms

#### 3.6.1. HLA-G

HLA-G are nonclassical class I tolerogenic molecules expressed as four membrane-bound (HLA-G1 and HLA-G4) and three secreted (HLA-5 and HLA-G7) isoforms. In the immune system, HLA-G are largely expressed by DCs and macrophages. HLA-G act through the inhibitory receptors ILT2 and ILT4 expressed by myeloid DCs, CD4^+^ and CD8^+^ T cells, B cells, monocytes/macrophages, and NK cells [107–109]. HLA-G inhibit alloproliferative T cell response, Th1 cell migration, Th1, and Th17 cytokine production, induce Treg cells, and suppress the cytotoxic function of NK cells [110–114]. This general pattern has been reported by several groups. However, the effect of HLA-G may depend on their concentration: in the study by Kapasi and coauthors, high doses of HLA-G promoted Tregs, whereas low doses fostered the development of Th1 cells [115].

Human MSCs express and secrete HLA-G5. The secretion of HLA-G5 by MSCs is stimulated by IL-10, IFN-*γ*, and TNF-*α* [112–114]. Myeloid APCs were shown to express HLA-G in pathological context (e.g., in cancer and viral infections); it was suggested that HLA-G-expressing myeloid APCs may be viewed as suppressor cells [108]. Yet, the role for HLA-G in the regulatory functions of MDSCs remains to be evaluated.

#### 3.6.2. CD39 and CD73

MSCs express ectonucleotidase CD73 that catabolizes AMP to adenosine. AMP is generated from ATP under the action of ectonucleoside CD39 that is expressed at low levels by MSCs and at high levels by activated T cells. Extracellular ATP exhibits proinflammatory effects; adenosine triggers inhibitory pathways mediated by cAMP and protein kinase A. Thus, the concerted action of CD39 and CD73 cleaves ATP to adenosine resulting in the immune suppression [116, 117]. Our search for data on the expression of CD39 and/or CD73 by MDSCs resulted in two original studies. One study reported the expression of CD73 by granulocytic MDSCs and the involvement of the nucleotidase activity in MDSCs-mediated suppression [118]. In another study, the anticancerogenic drug *α*-difluoromethylornithine hampered MDSC suppressive activity, in particular, by inhibiting the CD39/CD73-mediated pathway [119].

#### 3.6.3. Galectins

Galectins (Gal), soluble glycan-binding proteins, bind to cell surface glycoproteins. MSCs express Gal-1 and Gal-9. Gal-1 inhibits tissue emigration of immunogenic DCs [120] and selectively binds to Th1 and Th17 cells inducing their apoptosis but does not affect Th2 cells [121, 122]. Gal-1 upregulates the expression of AhR in T cells and the production of IL-10 by Th1 and Th17 cells [122]. Gal-9 mediates antiproliferative effects on T and B cells. In B lymphocytes, it also reduces immunoglobulin release. Gal-9 is upregulated by IFN-*γ* [123]. We found no reports on the usage of galectins by MDSCs in the available literature. However, galectins were shown to participate in the induction and the accumulation of MDSCs at tumor site [124].

#### 3.6.4. CCL2

The chemokine CCL2 interacts with CCR2 receptor expressed by myeloid cells and NK cells, activated Th1 and Th17 cells, and recruits them to the site of inflammation. MSCs produce CCL2 and express metalloproteinase that truncates CCL2, generating CCR2 antagonist that suppresses the migration of inflammatory cells. This mechanism seems to be critical for MSC-mediated suppression during autoimmune disorders. Defects in CCL2 processing have been associated with the pathogenesis of SLE [125]. In EAE, adoptively transferred wild-type MSCs induced immune suppression, whereas CCL2^−/−^ MSCs did not [126]. We found no reports on the usage of CCL2-mediated mechanism by MDSCs. However, MDSCs express CCR2 and readily respond to CCL2 by accumulating at the corresponding inflammatory sites [127].

#### 3.6.5. B7-H1

MSCs and MDSCs express negative costimulatory molecules, in particular, B7-H1. B7-H1 interacts with PD-1 [128]. The expression of B7-H1 by MSCs was induced by IFN-*γ* [129], whereas on MDSCs it could be induced by IL-13 [37]. Whether these differences are due to different experimental settings or are characteristic for MSCs and MDSCs remains to be clarified.

## 4. Cellular Targets

**Figure 2:**
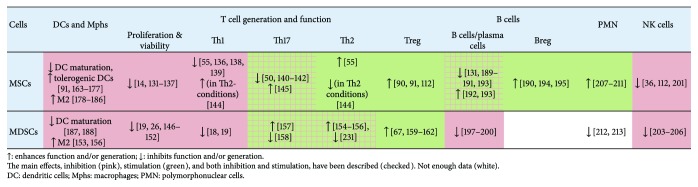
Cellular targets and modulatory effects of MSCs and MDSCs.

This section reviews immunomodulatory effects of MSCs and MDSCs on different immune cells (Figure 2).

### 4.1. T Lymphocytes

Effector T lymphocytes generate after naïve T cells recognize antigen, activate, proliferate, and differentiate into effector subsets. MSCs and MDSCs interfere with T cells at different stages of their differentiation and function.

#### 4.1.1. MSCs

MSCs hamper antigenic presentation by DCs and thus interfere with the antigenic stimulation of T cells, both* in vitro* and* in vivo* (see below). Activation of T cells, measured by the expression of CD69 and CD25, was inhibited by MSCs in some [130] but not in all [131, 132] studies. MSCs readily suppress T cell proliferation induced by mitogens, anti-CD3/CD28 stimulation, or transplantation antigens in MLR [14, 131–133]. The inhibitory effect is due to cell arrest in G0/G1 phase of cell cycle [131] and can be mediated by iNOS, PGE2 [134], IDO [135], TGF-*β* [14, 130], IL-10 [136], or PD-1 [134, 137]. The role for these molecular mediators in the suppression of T cell proliferation varies in different experimental settings. For example, the inhibition depended on IDO in some studies [135] but was IDO-independent in others [134, 137].

Functional activity of various T helper subsets is differentially affected by MSCs. Th1 and the production of IFN-*γ* are inhibited by MSCs through the production of PGE2 [55], IL-10 [136], IDO [138], cell contacts, and other mechanisms [139]. MSCs also suppress the generation of Th17, the expression of RORc in differentiating cells, and the production of IL-17 and IL-22 by Th17. The effects are mediated by PGE2 [50, 140, 141], IDO [50], and IL-10 [142].

MSCs do not suppress Th2 proliferation [138], stimulate the production of IL-4, and may switch from Th1 to Th2 response augmenting the production of IL-4, IL-10, and IL-13, supposedly through the PGE2-dependent mechanism [55].

MSCs promote the generation of Treg and enhance their activity and IL-10 production. The effect is mediated by TGF-*β* [90], by HLA-G5 [112], and indirectly through the generation of tolerogenic DCs (reviewed in detail in [91]).

This pattern is characteristic for MSCs derived from various sources and examined at different experimental settings. However, several exemptions have been reported. MSCs promoted the survival of quiescent T cells [143]. In Th2-predominating conditions, MSCs inhibited IL-4 and IL-5 and increased the production of IFN-*γ* and IL-2 [144]. BM-MSCs derived from rheumatoid arthritis and osteoarthritis patients induced the activation and the expansion of Th17 [145]. Dysfunction of MSCs has been associated with several autoimmune disorders [125, 145].

#### 4.1.2. MDSCs

In general, there are less data on the regulatory properties of MDSCs compared to MSCs. MDSCs inhibit the proliferation, IL-2, and IFN-*γ* production by T cells stimulated* in vitro* with anti-CD3/CD28, specific antigens, or in MLR [146–151]. The suppression is mediated through the production IL-10 [150], NO, and peroxynitrite [19, 26, 148, 151], and ARG1 [151, 152] and indirectly through the formation of M2 [153].

Data on the effects of MDSCs on Th2 and Th17 cells are limited. Several studies have reported that endogenous or adoptively transferred MDSCs increase Th2 response and inhibit graft-versus-host disease (GVHD) [154–156]. Both, promotion and suppression of Th17 by MDSCs have been shown [157, 158].

MDSCs promote* de novo* development of Foxp3+ Tregs* in vivo*. Different studies have associated this effect with the production of ARG-1 [67], IDO [159], IL-10 [160], CD40, and direct MDSC-Treg contacts [161, 162].

Comparison of the effects, which MSCs and MDSCs exert on T cells, shows similarities in (i) the inhibition of T cell proliferation and Th1 responses and (ii) the stimulation of Treg cells (Figure 2). This pattern corresponds to the mode of action of molecular mediators produced by MSCs and MDSCs (Figure 1). Th17 are generally suppressed by MSCs, although there are exemptions. Data on MDSCs-Th17 interactions are limited and contradictory. In line with this, different molecular mediators, utilized by MSCs and MDSCs, affect Th17 in different ways, suggesting that the final effect may depend on the combination of mediators that the cells produce in a giving experimental setting. The same is likely true for Th2 cells.

As discussed above, most of the mediators produced by MSCs and MDSCs are induced by proinflammatory type 1 cytokines (e.g., IFN-*γ*). This suggests that the cells play immunoregulatory role and control Th1 responses through the negative feedback loop. On the other hand, several mediators (i.e., ARG-1, TGF-*β*, and HLA-G5) can be induced by type 2 and regulatory cytokines (i.e., IL-13, IL-4, IL-10, and TGF-*β*). Whether in these “type 2 conditions” MSCs and MDSCs inhibit Th1 and support Th2 responses in a positive feedback manner, or switch their activity towards the suppression of Th2 (as it was demonstrated by Cho and coauthors [144]), is not completely clear. Further complication comes from the observations that the same mediator may play stimulatory or suppressive role depending on its concentration [44, 115] and that mediators produced by MSCs/MDSCs influence each other (see Figure 1). Evidently, studies are needed to create a quantitative model of cellular and molecular interactions that determine the final immunoregulatory properties of MSCs and MDSCs.

### 4.2. DCs and Macrophages

#### 4.2.1. MSCs

MSCs suppress monocyte differentiation into DCs, decrease the expression of MHC class II, CD80, CD86, CD83, and CD40 by DCs, lower DC capacity for endocytosis, suppress the production of IL-12 and TNF-*α* by DC type 1, and stimulate the production of IL-10 by DC type 2. Overall, MSCs inhibit antigen presentation and T cell stimulation and promote the generation of tolerogenic DCs [163–170]. These effects have been attributed to the production of PGE2 [166], IL-6 [164, 167], IL-10 [168, 171], HGF [104, 165, 172], and TNF-stimulated gene 6 protein (TSG-6) [169]. Many of these factors operate by activating JAK/STAT pathway and suppressing the activation of mitogen-activated protein kinases (MAPKs) and NF-*κ*B signaling pathways within DCs responding to TLR4 stimulation [168, 169, 173, 174]. Direct MSCs-DC contacts inhibit DC maturation and induce their tolerization by activating the Notch pathway [175] and altering actin cytoskeleton in the DCs [176].* In vivo* administration of MSCs decreased DC migration to the draining lymph node and hampered local CD4 T cell priming. The effect was attributed to the inhibition of MyD88 and the impairment of MAPKs and NF-*κ*B signaling pathways within DCs after TLR4 stimulation [177].

Two main and opposite types of macrophages have been defined, classically activated inflammatory (M1) and alternatively activated anti-inflammatory (M2) [178]. MSCs inhibit M1 and stimulate the generation of M2 macropahges: coculture of MSCs with BM-derived macrophages decreased the expression of iNOS, TNF-*α*, IL-6, IL-12, and CCL2 (i.e., the markers of M1) and upregulated the expression of IL-10, ARG-1, CD206, and STAT3 (i.e., the markers of M2) [179, 180]. Similar effects were observed* in vivo* [181]. The underlying factors were PGE2 [181], TSG-6 [182], IDO [183], IL-6 [184], and direct cell contacts.

The activation of M2 likely plays a role in the therapeutic effects of MSCs. In experimental settings, systemically infused gingival MSCs homed to the wound site, promoted host macrophage differentiation into M2, and enhanced wound repair [184]. In the models of acute lung injury, MSCs shifted macrophage phenotype from M1 to M2 attenuating lung tissue inflammation [185, 186]. In one study, the effect was partially due to insulin-like growth factor I (IGF-I) secreted by MSCs [186].

#### 4.2.2. MDSCs

MDSCs are cells that in the presence of appropriate cytokines differentiate into mature DCs and macrophages [6]. However, in pathology this differentiation is inhibited and MDSCs accumulate to affect different branches of immune response. Information on MDSCs-DCs and MDSCs-macrophages interactions is limited. In most studies, the effects were similar to those mediated by MSCs; that is, MDSCs inhibited DC maturation and polarized macrophages towards M2 phenotype, largely through the production of IL-10. In a clinical vaccine study, MDSCs copurified with monocytes suppressed DC maturation, antigen uptake, migration, and Th1 induction in a dose-dependent manner [187]. MDSCs isolated from hepatocellular carcinoma mice decreased the production of IL-12 by DCs [188].

When cocultured with macrophages, MDSCs reduced macrophage expression of MHC class II [17]. MDSCs from mice with spontaneous metastatic 4T1 mouse mammary carcinoma skewed macrophages towards the generation of M2 through IL-10 and cell contact dependent mechanisms [153]. In patients with esophageal cancer, increased ratio of MDSCs and augmented plasma levels of ARG1 were associated with high tissue expression of CD163, decreased IL-12 and IFN-*γ*, and increased GATA3, IL-4, IL-13, and IL-6 expressions (evidences of M2 polarization) [156].

In tumor environment, MDSCs can directly differentiate into suppressive tumor-associated macrophages (TAM) [6]. Of note, suppressive macrophages were shown to stimulate MDSCs for the production of IL-10 [17]. Thus, MDSCs may form a positive feedback loop with TAM.

### 4.3. B Cells

#### 4.3.1. MSCs

Data on MSCs-B lymphocyte interactions is not uniform. In some studies, MSCs inhibited proliferation, plasma-cell differentiation, IgM, IgG, and IgA production by B cells stimulated* in vitro* with CpG, recombinant CD40L, anti-Ig antibodies or IL-2, IL-4, and IL-10 cytokines. MSCs also suppressed B cell expression of CXCR4, CXCR5, and CCR7 and reduced B cell chemotaxis [131, 189–191].

In other studies, MSCs did not affect B cell proliferation [138] or even augmented it. In the study by Traggiai and coauthors, MSCs enhanced the proliferation of purified B cells obtained from healthy donors or pediatric systemic lupus erythematous (SLE) patients and their differentiation into plasma cells [192]. One possible explanation for these discrepancies comes from the study by Rasmusson and coauthors. The study suggests that MSCs' activity depends on the level of basal B cell response: MSCs reduced high-level IgG response and enhanced low-level production of IgG induced by LPS, cytomegalovirus, or varicella zoster virus, that is, played a homeostatic role [193].

Breg cells are induced by MSCs. The induction has been registered both* in vitro* and* in vivo* and resulted in the amelioration of GVHD [190, 194, 195].

Mechanisms underlying MSCs-B-cell interactions are complex and not fully understood. Different authors reported the involvement of IDO [195], MSC-T-cell contacts [190, 191], IL-10, soluble factor other than IDO, TGF-*β* or IL-10 [196], Galectin-9 [123], and CCL2 [125]. In SLE patients, BM-MSCs had reduced production of CCL2, which was associated with their defective capacity to suppress B cells. These findings suggest a potential role for MSCs in disease pathogenesis and demonstrate that MSCs generated in healthy and pathological conditions can exhibit different properties, uncovering another potential cause for conflicting data on MSCs-B cell interactions.

#### 4.3.2. MDSCs

Data on MDSCs-B lymphocytes interactions are highly limited and only start to accumulate. Most of existing data report inhibitory effect of MDSCs on B lymphocytes. Following murine retroviral LP-BM5 infection, MDSCs expanded and suppressed* ex vivo* B cell responses, partially through iNOS/NO- and VISTA-mediated mechanisms [197, 198]. MDSCs generated in the presence of adipocyte-conditioned medium inhibited B lymphopoiesis largely through IL-1 [199]. MDSCs from mice with collagen-induced arthritis inhibited autologous B cell proliferation and antibody production in NO, PGE2, and cell-cell contact dependent manner [200]. Administration of monocytic MDSCs reduced autoantibody production and rescued CCR2^−/−^ mice from the exacerbated collagen-induced arthritis [125, 200].

### 4.4. NK Cells

#### 4.4.1. MSCs

MSCs inhibit NK cell proliferation, expression of activating receptors, and decrease NK cytotoxicity and IFN-*γ* production [36]. In different settings, the effects were mediated by IDO, PGE2, TGF-*β*, HLA-5, and cell contacts [36, 112, 201]. Following their coculture with MSCs, NK upregulate the expression of CD73 that has anti-inflammatory effect [202].

#### 4.4.2. MDSCs

NK cultured with MDSCs produce less IFN-*γ*. The suppression has been attributed to the production of ARG1 [203], COX2/PGE2 [204], cell-cell contacts involving NK cell activation receptor NKG2D, and membrane-bound TGF*β* [205]. The role for MDSCs in the inhibition of NK* in vivo* was demonstrated in the study by Zhu and coauthors, who described the generation of granulocytic MDSCs following the administration of adenoviral vectors in mice; depletion of MDSCs enhanced NK responses and accelerated virus clearance [206].

### 4.5. Neutrophils

Neutrophils are nonproliferating short-living cells that rapidly migrate to the site of infection/inflammation and eliminate pathogens or cellular debris.

#### 4.5.1. MSCs

MSCs generally exhibit proneutrophilic action supporting neutrophil survival and inhibiting their apoptosis. The proneutrophilic effect was demonstrated for MSCs derived from various sources (i.e., BM, glandular, and adipose tissue) and was largely mediated by IL-6 [207]. It has been suggested that the proneutrophilic effect of MSCs plays a role in supporting this short-living population in the BM. MSCs activated by LPS stimulate the expression of CD11b by neutrophils [208] and are able to recruit neutrophils in IL-8 and MIF-dependent manner [209]. Data on the influence of MSCs on antibacterial properties of neutrophils are not uniform. In some studies, BM-MSCs dampened neutrophil respiratory burst [207], while in others enhanced it [208]. The stimulatory effect depended on IL-6, IFN-*β*, and GM-CSF [208]. Hall and coauthors have demonstrated that MSCs may affect neutrophil function* in vivo*: the administration of BM-MSCs to septic mice stimulated bacteria clearance; neutrophil depletion abrogated the effect [210]. In one study, proneutrophilic effect of MSCs was mediated through the induction of Th17 [211].

#### 4.5.2. MDSCs

The influence of MDSCs on neutrophils remains underinvestigated. Existing data are largely limited by the observation that the accumulation of MDSCs is accompanied by gradual disappearance of mature neutrophils [19]. These opposing relationships between MDSCs and neutrophils may be due to the incomplete differentiation of immature myeloid cells in inflammatory conditions leading to the accumulation of MDSCs and simultaneously to the reduction of mature neutrophils. Another suggested mechanism is efferocytosis of apoptotic neutrophils by MDSCs, which has been described in mice infected with* Klebsiella pneumoniae* or challenged with LPS [212, 213]. Thus, differentially from MSCs, MDSCs and neutrophils seem to be mutually exclusive. However, this speculation needs further investigation.

### 4.6. Interactions between MSCs and MDSCs

Only few studies directly addressed the interplay between MSCs and MDSCs. In the study by Yen and coauthors, human MSCs expanded CD14^−^CD11b^+^CD33^+^ MDSCs that expressed ARG1 and NO, suppressed lymphocyte proliferation, and promoted Treg generation. The effect was mediated through the secretion of HGF and the induction of STAT3 [78]. In another study, growth-regulated oncogene GRO-*γ* secreted by MSCs suppressed the generation of monocyte-derived DCs and stimulated the formation of MDSCs. The latter secreted IL-10 and IL-4 and expressed ARG1 and iNOS [214]. Galectins, known to be produced by MSCs, were reported to participate in the expansion of MDSCs at tumor sites [124]. MDSCs produce ROS. In physiological levels, ROS support MSCs' proliferation and differentiation, and, in higher amount, ROS promote MSCs' aging [84]. Other mediators produced by MSCs and MDSCs (e.g., PGE2) can activate both populations of cells in a positive feedback manner.

## 5. Concluding Remarks

In this review, we have compared mechanisms and modes of immunoregulatory action of two immature cell populations: MSCs and MDSCs. The populations belong to two distinct differentiation lineages, but both are able to regulate immune response. MSCs and MDSCs share many immunomodulatory mechanisms and exert similar effects. In particular, they inhibit DC and macrophage maturation, antigen presentation, and suppress T cell proliferation, Th1 responses and NK activity. Both populations promote the generation of tolerogenic DCs, M2 macrophages, and regulatory T cells. Proinflammatory conditions activate suppressor capacities of both MSCs and MDSCs. This likeness is largely due to the usage of similar set of mediators, for example, IDO, PGE2, IL-10, and TGF-*β* (Figure 1).

In spite of these similarities, comparative analysis reveals the discrepancies between the two populations. First, some mediators are produced by one but not by another subset. In particular, ARG1 and ROS seem to be restricted to MDSCs; the production of galectins and HLA-G has been attributed to MSCs but not to MDSCs. Second, MSCs and MDSCs are generally activated by proinflammatory type 1 cytokines. However, in some conditions, they can be stimulated by type 2 cytokines, and MDSCs seem to be more prone to this type of stimulation. Indeed, ARG1, which is expressed in MDSCs but not in MSCs, can be induced by IL-4, IL-13, and TGF-*β*. In MSCs, the expression of B7-H1 and the production of TGF-*β* were induced by IFN-*γ*, whereas in MDSCs by IL-10 and IL-13 [37, 129]. Third, MSCs exert proneutrophilic effects, supporting neutrophil survival and function [207–210]. MDSCs, in contrast, seem to oppose neutrophilic inflammation [19, 212, 213]. Fourth, MSCs expand MDSCs. Whether and how MDSCs affect MSCs is largely unknown. Fifth, MSCs are generally considered as immunoregulatory cells that can inhibit or enhance immune function depending on cell microenvironment [5, 15]. MDSCs are currently regarded as immunosuppressive cells. Whether MDSCs may be considered as immunoregulatory cells that act by supporting immune homeostasis is not yet clear.

Several other questions remain unanswered and need further investigation.

In particular, the pattern of the interplay between MSCs/MDSCs and Th2, Th17, and B lymphocytes is not fully clear. Both stimulation and inhibition of these responses by MSCs and MDSCs have been documented [55, 144, 154–156, 189–193, 231], and exact factors that determine the ultimate result are yet to be determined. One group of factors is represented by TLR ligands. The contribution of different TLR-mediated pathways to pro- or anti-inflammatory functions of MSCs/MDSCs is one of the recently emerged areas of research [213, 215].

In proinflammatory conditions, MSCs and MDSCs are activated to inhibit type 1 response, that is, act in a negative feedback manner. Whether in “type 2 conditions” the cells inhibit Th1 or Th2 responses, that is, participate in positive or negative feedback loop, remains unclear.

As noted above, MSCs and MDSCs share a set of core regulatory mediators and mechanisms. However, they differentially affect some immune cells. Molecular mechanisms underlying these discrepancies remain unknown.

MSCs and MDSC can simultaneously produce a wide range of immunoregulatory factors that have similar but not identical activity (Figure 1). Furthermore, the subsets of the produced factors and the amounts secreted may vary in different conditions. Overall, this creates the possibility for MSCs/MDSCs to fine-tune different branches of the immune response and simultaneously makes their final effect difficult to predict. Quantitative models of cellular and molecular interactions that determine the final immunoregulatory properties of MSCs and MDSCs would help to predict their effects in various microenvironments, both* in vitro* and* in vivo.*

Speaking about possible clinical applications, MSCs are widely considered for the purposes of clinical immunomodulation due to their homeostatic properties and the feasibility of generating the large numbers of autologous cells. MSCs have been suggested as a mean to treat severe life-threatening forms of autoimmune and autoinflammatory diseases (e.g., SLE, systemic sclerosis, and inflammatory bowel disease [216–219]), prevent and treat steroid-refractory graft-versus-host disease [220], improve the outcome after organ transplantation [221], and stimulate tissue repair, regeneration, and wound healing [222–227]. Detailed analysis of MSC therapeutic potential, risks, and limitations of their application is beyond the focus of the current review. In contrast to MSCs, MDSCs are usually regarded as the target for immunomodulation, particularly, in cancer where they accumulate abundantly and contribute to pathology [228, 229]. However, in autoimmune pathology, MDSC dysfunction may be a factor driving disease progression and can be limited by the administration of exogenous MDSCs [24]. Thus, the question whether MDSCs can be used for therapeutic immunomodulation in some pathological conditions remains to be explored. It is important to note that MDSCs can be grown* in vitro* and they are more differentiated compared to MSCs; thus, they have a lower risk of cell transformation.

In conclusion, this review does not imply to describe all effects and mechanisms mediated by MSCs/MDSCs, as they are multiple and vary in different conditions. Rather, it is an attempt to compare the main patterns of MSCs/MDSCs' activities in a way to detect cell similarities and discrepancies and identify new directions for their investigation.

## References

[1] Vignali D. A. A., Collison L. W., Workman C. J. (2008). How regulatory T cells work. *Nature Reviews Immunology*.

[2] Benoist C., Mathis D. (2012). Treg cells, life history, and diversity. *Cold Spring Harbor Perspectives in Biology*.

[3] Durand J., Chiffoleau E. (2015). B cells with regulatory properties in transplantation tolerance. *World Journal of Transplantation*.

[4] Liu J., Chen D., Nie G. D., Dai Z. (2015). CD8^+^CD122^+^ T-cells: a newly emerging regulator with central memory cell phenotypes. *Frontiers in Immunology*.

[5] Najar M., Raicevic G., Fayyad-Kazan H., Bron D., Toungouz M., Lagneaux L. (2016). Mesenchymal stromal cells and immunomodulation: a gathering of regulatory immune cells. *Cytotherapy*.

[6] Gabrilovich D. I., Nagaraj S. (2009). Myeloid-derived suppressor cells as regulators of the immune system. *Nature Reviews Immunology*.

[7] Glenn J. D., Whartenby K. A. (2014). Mesenchymal stem cells: emerging mechanisms of immunomodulation and therapy. *World Journal of Stem Cells*.

[8] Dazzi F., Ramasamy R., Glennie S., Jones S. P., Roberts I. (2006). The role of mesenchymal stem cells in haemopoiesis. *Blood Reviews*.

[9] Ehninger A., Trumpp A. (2011). The bone marrow stem cell niche grows up: mesenchymal stem cells and macrophages move in. *Journal of Experimental Medicine*.

[10] Isern J., Martín-Antonio B., Ghazanfari R. (2013). Self-renewing human bone marrow mesenspheres promote hematopoietic stem cell expansion. *Cell Reports*.

[11] Dominici M., Le Blanc K., Mueller I. (2006). Minimal criteria for defining multipotent mesenchymal stromal cells. The International Society for Cellular Therapy position statement. *Cytotherapy*.

[12] Boxall S. A., Jones E. (2012). Markers for characterization of bone marrow multipotential stromal cells. *Stem Cells International*.

[13] Mabuchi Y., Houlihan D. D., Akazawa C., Okano H., Matsuzaki Y. (2013). Prospective isolation of murine and human bone marrow mesenchymal stem cells based on surface markers. *Stem Cells International*.

[14] Di Nicola M., Carlo-Stella C., Magni M. (2002). Human bone marrow stromal cells suppress T-lymphocyte proliferation induced by cellular or nonspecific mitogenic stimuli. *Blood*.

[15] Mattar P., Bieback K. (2015). Comparing the immunomodulatory properties of bone marrow, adipose tissue, and birth-associated tissue mesenchymal stromal cells. *Frontiers in Immunology*.

[16] Condamine T., Gabrilovich D. I. (2011). Molecular mechanisms regulating myeloid-derived suppressor cell differentiation and function. *Trends in Immunology*.

[17] Ostrand-Rosenberg S., Sinha P. (2009). Myeloid-derived suppressor cells: linking inflammation and cancer. *The Journal of Immunology*.

[18] Cuenca A. G., Delano M. J., Kelly-Scumpia K. M. (2011). A paradoxical role for myeloid-derived suppressor cells in sepsis and trauma. *Molecular Medicine*.

[19] Tsiganov E. N., Verbina E. M., Radaeva T. V. (2014). Gr-1dimCD11b+ immature myeloid-derived suppressor cells but not neutrophils are markers of lethal tuberculosis infection in mice. *Journal of Immunology*.

[20] Haile L. A., Gamrekelashvili J., Manns M. P., Korangy F., Greten T. F. (2010). CD49d is a new marker for distinct myeloid-derived suppressor cell subpopulations in mice. *The Journal of Immunology*.

[21] Dilek N., Vuillefroy de Silly R., Blancho G., Vanhove B. (2012). Myeloid-derived suppressor cells: mechanisms of action and recent advances in their role in transplant tolerance. *Frontiers in Immunology*.

[22] Marvel D., Gabrilovich D. I. (2015). Myeloid-derived suppressor cells in the tumor microenvironment: expect the unexpected. *The Journal of Clinical Investigation*.

[23] Ortiz M. L., Lu L., Ramachandran I., Gabrilovich D. I. (2014). Myeloid-derived suppressor cells in the development of lung cancer. *Cancer Immunology Research*.

[24] Cripps J. G., Gorham J. D. (2011). MDSC in autoimmunity. *International Immunopharmacology*.

[25] Van Ginderachter J. A., Beschin A., Baetselier P. D., Raes G. (2010). Myeloid-derived suppressor cells in parasitic infections. *European Journal of Immunology*.

[26] Goño O., Alcaide P., Fresno M. (2002). Immunosuppression during acute *Trypanosoma cruzi* infection: involvement of Ly6G (Gr1^+^)CD11b^+^ immature myeloid suppressor cells. *International Immunology*.

[27] Soliman H., Mediavilla-Varela M., Antonia S. (2010). Indoleamine 2,3-dioxygenase is it an immune suppressor?. *Cancer Journal*.

[28] Munn D. H., Mellor A. L. (2013). Indoleamine 2,3 dioxygenase and metabolic control of immune responses. *Trends in Immunology*.

[29] Sharma M. D., Hou D.-Y., Liu Y. (2009). Indoleamine 2,3-dioxygenase controls conversion of Foxp3^+^ Tregs to TH17-like cells in tumor-draining lymph nodes. *Blood*.

[30] Sundrud M. S., Koralov S. B., Feuerer M. (2009). Halofuginone inhibits th17 cell differentiation by activating the amino acid starvation response. *Science*.

[31] Brenk M., Scheler M., Koch S. (2009). Tryptophan deprivation induces inhibitory receptors ILT3 and ILT4 on dendritic cells favoring the induction of human CD4^+^CD25^+^ Foxp3^+^ T regulatory cells. *Journal of Immunology*.

[32] Xu H., Zhang G.-X., Ciric B., Rostami A. (2008). IDO: a double-edged sword for T_H_1/T_H_2 regulation. *Immunology Letters*.

[33] Xu H., Oriss T. B., Fei M. (2008). Indoleamine 2,3-dioxygenase in lung dendritic cells promotes Th2 responses and allergic inflammation. *Proceedings of the National Academy of Sciences of the United States of America*.

[34] Nguyen N. T., Kimura A., Nakahama T. (2010). Aryl hydrocarbon receptor negatively regulates dendritic cell immunogenicity via a kynurenine-dependent mechanism. *Proceedings of the National Academy of Sciences of the United States of America*.

[35] Ryan J. M., Barry F., Murphy J. M., Mahon B. P. (2007). Interferon-*γ* does not break, but promotes the immunosuppressive capacity of adult human mesenchymal stem cells. *Clinical and Experimental Immunology*.

[36] Spaggiari G. M., Capobianco A., Abdelrazik H., Becchetti F., Mingari M. C., Moretta L. (2008). Mesenchymal stem cells inhibit natural killer-cell proliferation, cytotoxicity, and cytokine production: role of indoleamine 2,3-dioxygenase and prostaglandin E2. *Blood*.

[37] Pinton L., Solito S., Damuzzo V. (2015). Activated T cells sustain myeloid-derived suppressor cell-mediated immune suppression. *Oncotarget*.

[38] Chon S. Y., Hassanain H. H., Gupta S. L. (1996). Cooperative role of interferon regulatory factor 1 and p91 (STAT1) response elements in interferon-*γ*-inducible expression of human indoleamine 2,3-dioxygenase gene. *The Journal of Biological Chemistry*.

[39] Dai W., Gupta S. L. (1990). Regulation of indoleamine 2,3-dioxygenase gene expression in human fibroblasts by interferon-*γ*. Upstream control region discriminates between interferon-*γ* and interferon-*α*. *Journal of Biological Chemistry*.

[40] Orabona C., Puccetti P., Vacca C. (2006). Toward the identification of a tolerogenic signature in IDO-competent dendritic cells. *Blood*.

[41] Yagi H., Soto-Gutierrez A., Parekkadan B. (2010). Mesenchymal stem cells: mechanisms of immunomodulation and homing. *Cell Transplantation*.

[42] Braun D., Longman R. S., Albert M. L. (2005). A two-step induction of indoleamine 2,3 dioxygenase (IDO) activity during dendritic-cell maturation. *Blood*.

[43] Rodríguez M., Domingo E., Municio C. (2014). Polarization of the innate immune response by prostaglandin E2: a puzzle of receptors and signals. *Molecular Pharmacology*.

[44] Sreeramkumar V., Fresno M., Cuesta N. (2012). Prostaglandin E2 and T cells: friends or foes?. *Immunology & Cell Biology*.

[45] Betz M., Fox B. S. (1991). Prostaglandin E2 inhibits production of Th1 lymphokines but not of Th2 lymphokines. *Journal of Immunology*.

[46] Meyer F., Ramanujam K. S., Gobert A. P., James S. P., Wilson K. T. (2003). Cutting edge: cyclooxygenase-2 activation suppresses Th1 polarization in response to *Helicobacter pylori*. *The Journal of Immunology*.

[47] Ylöstalo J. H., Bartosh T. J., Coble K., Prockop D. J. (2012). Human mesenchymal stem/stromal cells cultured as spheroids are self-activated to produce prostaglandin E2 that directs stimulated macrophages into an anti-inflammatory phenotype. *Stem Cells*.

[48] Khayrullina T., Yen J.-H., Jing H., Ganea D. (2008). In vitro differentiation of dendritic cells in the presence of prostaglandin E2 alters the IL-12/IL-23 balance and promotes differentiation of Thl7 cells. *Journal of Immunology*.

[49] Yao C., Sakata D., Esaki Y. (2009). Prostaglandin E2-EP4 signaling promotes immune inflammation through T_H_1 cell differentiation and T_H_17 cell expansion. *Nature Medicine*.

[50] Tatara R., Ozaki K., Kikuchi Y. (2011). Mesenchymal stromal cells inhibit Th17 but not regulatory T-cell differentiation. *Cytotherapy*.

[51] Sharma S., Yang S.-C., Zhu L. (2005). Tumor cyclooxygenase-2/prostaglandin E_2_-dependent promotion of FOXP3 expression and CD4^+^CD25^+^ T regulatory cell activities in lung cancer. *Cancer Research*.

[52] Fujita M., Kohanbash G., Fellows-Mayle W. (2011). COX-2 blockade suppresses gliomagenesis by inhibiting myeloid-derived suppressor cells. *Cancer Research*.

[53] Obermajer N., Kalinski P. (2012). Generation of myeloid-derived suppressor cells using prostaglandin E_2_. *Transplantation Research*.

[54] Mao Y., Sarhan D., Steven A., Seliger B., Kiessling R., Lundqvist A. (2014). Inhibition of tumor-derived prostaglandin-E2 blocks the induction of myeloid-derived suppressor cells and recovers natural killer cell activity. *Clinical Cancer Research*.

[55] Aggarwal S., Pittenger M. F. (2005). Human mesenchymal stem cells modulate allogeneic immune cell responses. *Blood*.

[56] Jarvinen L., Badri L., Wettlaufer S. (2008). Lung resident mesenchymal stem cells isolated from human lung allografts inhibit T cell proliferation via a soluble mediator. *The Journal of Immunology*.

[57] Eruslanov E., Daurkin I., Ortiz J., Vieweg J., Kusmartsev S. (2010). Pivotal advance: tumor-mediated induction of myeloid-derived suppressor cells and M2-polarized macrophages by altering intracellular PGE2 catabolism in myeloid cells. *Journal of Leukocyte Biology*.

[58] Serafini P. (2010). Editorial: PGE2-producing MDSC: a role in tumor progression?. *Journal of Leukocyte Biology*.

[59] English K., Barry F. P., Field-Corbett C. P., Mahon B. P. (2007). IFN-*γ* and TNF-*α* differentially regulate immunomodulation by murine mesenchymal stem cells. *Immunology Letters*.

[60] Rodriguez P. C., Quiceno D. G., Ochoa A. C. (2007). L-arginine availability regulates T-lymphocyte cell-cycle progression. *Blood*.

[61] Rodriguez P. C., Zea A. H., Culotta K. S., Zabaleta J., Ochoa J. B., Ochoa A. C. (2002). Regulation of T cell receptor *CD*3*ζ* chain expression by l-arginine. *The Journal of Biological Chemistry*.

[62] Modolell M., Choi B.-S., Ryan R. O. (2009). Local suppression of T cell responses by arginase-induced L-arginine depletion in nonhealing leishmaniasis. *PLoS Neglected Tropical Diseases*.

[63] Herbert D. R., Orekov T., Roloson A. (2010). Arginase I suppresses IL-12/IL-23p40-driven intestinal inflammation during acute schistosomiasis. *Journal of Immunology*.

[64] El Kasmi K. C., Qualls J. E., Pesce J. T. (2008). Toll-like receptor-induced arginase 1 in macrophages thwarts effective immunity against intracellular pathogens. *Nature Immunology*.

[65] Yang M., Rangasamy D., Matthaei K. I. (2006). Inhibition of arginase I activity by RNA interference attenuates IL-13-induced airways hyperresponsiveness. *The Journal of Immunology*.

[66] Pesce J. T., Ramalingam T. R., Mentink-Kane M. M. (2010). Arginase-1-expressing macrophages suppress Th2 cytokine-driven inflammation and fibrosis. *PLoS Pathogens*.

[67] Serafini P., Mgebroff S., Noonan K., Borrello I. (2008). Myeloid-derived suppressor cells promote cross-tolerance in B-cell lymphoma by expanding regulatory T cells. *Cancer Research*.

[68] Chang J., Thangamani S., Kim M. H., Ulrich B., Morris S. M., Kim C. H. (2013). Retinoic acid promotes the development of Arg1-expressing dendritic cells for the regulation of T-cell differentiation. *European Journal of Immunology*.

[69] Bingisser R. M., Tilbrook P. A., Holt P. G., Kees U. R. (1998). Macrophage-derived nitric oxide regulates T cell activation via reversible disruption of the Jak3/STAT5 signaling pathway. *The Journal of Immunology*.

[70] Mazzoni A., Bronte V., Visintin A. (2002). Myeloid suppressor lines inhibit T cell responses by an NO-dependent mechanism. *Journal of Immunology*.

[71] Harari O., Liao J. K. (2004). Inhibition of MHC II gene transcription by nitric oxide and antioxidants. *Current Pharmaceutical Design*.

[72] Taylor-Robinson A. W., Liew F. Y., Severn A. (1994). Regulation of the immune response by nitric oxide differentially produced by T helper type 1 and T helper type 2 cells. *European Journal of Immunology*.

[73] Bauer H., Jung T., Tsikas D., Stichtenoth D. O., Frölich J. C., Neumann C. (1997). Nitric oxide inhibits the secretion of T-helper 1- and T-helper 2-associated cytokines in activated human T cells. *Immunology*.

[74] Munder M., Mollinedo F., Calafat J. (2005). Arginase I is constitutively expressed in human granulocytes and participates in fungicidal activity. *Blood*.

[75] Gordon S. (2003). Alternative activation of macrophages. *Nature Reviews Immunology*.

[76] Zea A. H., Rodriguez P. C., Atkins M. B. (2005). Arginase-producing myeloid suppressor cells in renal cell carcinoma patients: a mechanism of tumor evasion. *Cancer Research*.

[77] Sato K., Ozaki K., Oh I. (2007). Nitric oxide plays a critical role in suppression of T-cell proliferation by mesenchymal stem cells. *Blood*.

[78] Yen B. L., Yen M.-L., Hsu P.-J. (2013). Multipotent human mesenchymal stromal cells mediate expansion of myeloid-derived suppressor cells via hepatocyte growth factor/c-Met and STAT3. *Stem Cell Reports*.

[79] Ren G., Zhang L., Zhao X. (2008). Mesenchymal stem cell-mediated immunosuppression occurs via concerted action of chemokines and nitric oxide. *Cell Stem Cell*.

[80] Han X., Yang Q., Lin L. (2014). Interleukin-17 enhances immunosuppression by mesenchymal stem cells. *Cell Death and Differentiation*.

[81] Talmadge J. E., Hood K. C., Zobel L. C., Shafer L. R., Coles M., Toth B. (2007). Chemoprevention by cyclooxygenase-2 inhibition reduces immature myeloid suppressor cell expansion. *International Immunopharmacology*.

[82] Lu T., Gabrilovich D. I. (2012). Molecular pathways: tumor-infiltrating myeloid cells and reactive oxygen species in regulation of tumor microenvironment. *Clinical Cancer Research*.

[83] Molon B., Ugel S., Del Pozzo F. (2011). Chemokine nitration prevents intratumoral infiltration of antigen-specific T cells. *The Journal of Experimental Medicine*.

[84] Yang S.-R., Park J.-R., Kang K.-S. (2015). Reactive oxygen species in mesenchymal stem cell aging: implication to lung diseases. *Oxidative Medicine and Cellular Longevity*.

[85] Wrzesinski S. H., Wan Y. Y., Flavell R. A. (2007). Transforming growth factor-*β* and the immune response: implications for anticancer therapy. *Clinical Cancer Research*.

[86] Heath V. L., Murphy E. E., Crain C., Tomlinson M. G., O'Garra A. (2000). TGF-*β*1 down-regulates Th2 development and results in decreased IL-4-induced STAT6 activation and GATA-3 expression. *European Journal of Immunology*.

[87] Gorelik L., Constant S., Flavell R. A. (2002). Mechanism of transforming growth factor *β*-induced inhibition of T helper type 1 differentiation. *The Journal of Experimental Medicine*.

[88] Evans C. M., Jenner R. G. (2013). Transcription factor interplay in T helper cell differentiation. *Briefings in Functional Genomics*.

[89] Xu C., Yu P., Han X. (2014). TGF-*β* promotes immune responses in the presence of mesenchymal stem cells. *The Journal of Immunology*.

[90] Melief S. M., Schrama E., Brugman M. H. (2013). Multipotent stromal cells induce human regulatory T cells through a novel pathway involving skewing of monocytes toward anti-inflammatory macrophages. *Stem Cells*.

[91] Engela A. U., Baan C. C., Dor F. J. M. F., Weimar W., Hoogduijn M. J. (2012). On the interactions between mesenchymal stem cells and regulatory T cells for immunomodulation in transplantation. *Frontiers in Immunology*.

[92] Fichtner-Feigl S., Terabe M., Kitani A. (2008). Restoration of tumor immunosurveillance via targeting of interleukin-13 receptor-*α*2. *Cancer Research*.

[93] Moore K. W., De Waal Malefyt R., Coffman R. L., O'Garra A. (2001). Interleukin-10 and the interleukin-10 receptor. *Annual Review of Immunology*.

[94] Hazlett L. D., Jiang X., Mcclellan S. A. (2014). IL-10 function, regulation, and in bacterial keratitis. *Journal of Ocular Pharmacology and Therapeutics*.

[95] Chaudhry A., Samstein R. M., Treuting P. (2011). Interleukin-10 signaling in regulatory T cells is required for suppression of Th17 cell-mediated inflammation. *Immunity*.

[96] Akbari O., DeKruyff R. H., Umetsu D. T. (2001). Pulmonary dendritic cells producing IL-10 mediate tolerance induced by respiratory exposure to antigen. *Nature Immunology*.

[97] Zdrenghea M. T., Makrinioti H., Muresan A., Johnston S. L., Stanciu L. A. (2015). The role of macrophage IL-10/innate IFN interplay during virus-induced asthma. *Reviews in Medical Virology*.

[98] Walter M. R. (2014). The molecular basis of IL-10 function: from receptor structure to the onset of signaling. *Current Topics in Microbiology and Immunology*.

[99] Brooks D. G., Walsh K. B., Elsaesser H., Oldstone M. B. A. (2010). IL-10 directly suppresses CD4 but not CD8 T cell effector and memory responses following acute viral infection. *Proceedings of the National Academy of Sciences of the United States of America*.

[100] Saraiva M., O'Garra A. (2010). The regulation of IL-10 production by immune cells. *Nature Reviews Immunology*.

[101] Harizi H., Gualde N. (2006). Pivotal role of PGE2 and IL-10 in the cross-regulation of dendritic cell-derived inflammatory mediators. *Cellular and Molecular Immunology*.

[102] Benkhoucha M., Santiago-Raber M.-L., Schneiter G. (2010). Hepatocyte growth factor inhibits CNS autoimmunity by inducing tolerogenic dendritic cells and CD25^+^Foxp3^+^ regulatory T cells. *Proceedings of the National Academy of Sciences of the United States of America*.

[103] Okunishi K., Dohi M., Fujio K. (2007). Hepatocyte growth factor significantly suppresses collagen-induced arthritis in mice. *Journal of Immunology*.

[104] Chen P.-M., Liu K.-J., Hsu P.-J. (2014). Induction of immunomodulatory monocytes by human mesenchymal stem cell-derived hepatocyte growth factor through ERK1/2. *Journal of Leukocyte Biology*.

[105] Madrigal M., Rao K. S., Riordan N. H. (2014). A review of therapeutic effects of mesenchymal stem cell secretions and induction of secretory modification by different culture methods. *Journal of Translational Medicine*.

[106] Toh B., Wang X., Keeble J. (2011). Mesenchymal transition and dissemination of cancer cells is driven by myeloid-derived suppressor cells infiltrating the primary tumor. *PLoS Biology*.

[107] LeMaoult J., Zafaranloo K., Le Banff C., Carosella E. D. (2005). HLA-G up-regulates ILT2, ILT3, ILT4, and KIR2DL4 in antigen presenting cells, NK cells, and T cells. *The FASEB Journal*.

[108] Carosella E. D., Gregori S., LeMaoult J. (2011). The tolerogenic interplay(s) among HLA-G, myeloid APCs, and regulatory cells. *Blood*.

[109] Curigliano G., Criscitiello C., Gelao L., Goldhirsch A. (2013). Molecular pathways: human leukocyte antigen G(HLA-G). *Clinical Cancer Research*.

[110] Morandi F., Ferretti E., Bocca P., Prigione I., Raffaghello L., Pistoia V. (2010). A novel mechanism of soluble HLA-G mediated immune modulation: downregulation of T cell chemokine receptor expression and impairment of chemotaxis. *PLoS ONE*.

[111] Agaugué S., Carosella E. D., Rouas-Freiss N. (2011). Role of HLA-G in tumor escape through expansion of myeloid-derived suppressor cells and cytokinic balance in favor of Th2 versus Th1/Th17. *Blood*.

[112] Selmani Z., Naji A., Zidi I. (2008). Human leukocyte antigen-G5 secretion by human mesenchymal stem cells is required to suppress T lymphocyte and natural killer function and to induce CD4^+^CD25^high^FOXP3^+^ regulatory T cells. *STEM CELLS*.

[113] Nasef A., Mathieu N., Chapel A. (2007). Immunosuppressive effects of mesenchymal stem cells: involvement of HLA-G. *Transplantation*.

[114] Montespan F., Deschaseaux F., Sensébé L., Carosella E. D., Rouas-Freiss N. (2014). Osteodifferentiated mesenchymal stem cells from bone marrow and adipose tissue express HLA-G and display immunomodulatory properties in Hla-mismatched settings: implications in bone repair therapy. *Journal of Immunology Research*.

[115] Kapasi K., Albert S. E., Yie S.-M., Zavazava N., Librach C. L. (2000). HLA-G has a concentration-dependent effect on the generation of an allo-CTL response. *Immunology*.

[116] Kerkelä E., Laitinen A., Räbinä J. (2016). Adenosinergic immunosuppression by human mesenchymal stromal cells (MSCs) requires co-operation with T cells. *Stem Cells*.

[117] Chen M., Su W., Lin X. (2013). Adoptive transfer of human gingiva-derived mesenchymal stem cells ameliorates collagen-induced arthritis via suppression of Th1 and Th17 cells and enhancement of regulatory T cell differentiation. *Arthritis and Rheumatism*.

[118] Ryzhov S., Novitskiy S. V., Goldstein A. E. (2011). Adenosinergic regulation of the expansion and immunosuppressive activity of CD11b^+^Gr1^+^ cells. *Journal of Immunology*.

[119] Ye C., Geng Z., Dominguez D. (2016). Targeting ornithine decarboxylase by *α*-difluoromethylornithine inhibits tumor growth by impairing myeloid-derived suppressor cells. *The Journal of Immunology*.

[120] Thiemann S., Man J. H., Chang M. H., Lee B., Baum L. G. (2015). Galectin-1 regulates tissue exit of specific dendritic cell populations. *The Journal of Biological Chemistry*.

[121] Toscano M. A., Bianco G. A., Ilarregui J. M. (2007). Differential glycosylation of TH1, TH2 and TH-17 effector cells selectively regulates susceptibility to cell death. *Nature Immunology*.

[122] Cedeno-Laurent F., Opperman M., Barthel S. R., Kuchroo V. K., Dimitroff C. J. (2012). Galectin-1 triggers an immunoregulatory signature in Th cells functionally defined by IL-10 expression. *The Journal of Immunology*.

[123] Ungerer C., Quade-Lyssy P., Radeke H. H. (2014). Galectin-9 is a suppressor of T and B cells and predicts the immune modulatory potential of mesenchymal stromal cell preparations. *Stem Cells and Development*.

[124] Verschuere T., Toelen J., Maes W. (2014). Glioma-derived galectin-1 regulates innate and adaptive antitumor immunity. *International Journal of Cancer*.

[125] Che N., Li X., Zhang L. (2014). Impaired B cell inhibition by lupus bone marrow mesenchymal stem cells is caused by reduced CCl2 expression. *Journal of Immunology*.

[126] Rafei M., Campeau P. M., Aguilar-Mahecha A. (2009). Mesenchymal stromal cells ameliorate experimental autoimmune encephalomyelitis by inhibiting CD4 Th17 T cells in a CC chemokine ligand 2-dependent manner. *The Journal of Immunology*.

[127] Hale M., Itani F., Buchta C. M., Wald G., Bing M., Norian L. A. (2015). Obesity triggers enhanced MDSC accumulation in murine renal tumors via elevated local production of CCL2. *PLoS ONE*.

[128] Augello A., Tasso R., Negrini S. M. (2005). Bone marrow mesenchymal progenitor cells inhibit lymphocyte proliferation by activation of the programmed death 1 pathway. *European Journal of Immunology*.

[129] Sheng H., Wang Y., Jin Y. (2008). A critical role of IFN*γ* in priming MSC-mediated suppression of T cell proliferation through up-regulation of B7-H1. *Cell Research*.

[130] Groh M. E., Maitra B., Szekely E., Koç O. N. (2005). Human mesenchymal stem cells require monocyte-mediated activation to suppress alloreactive T cells. *Experimental Hematology*.

[131] Glennie S., Soeiro I., Dyson P. J., Lam E. W.-F., Dazzi F. (2005). Bone marrow mesenchymal stem cells induce division arrest anergy of activated T cells. *Blood*.

[132] Nagaya R., Mizuno-Kamiya M., Takayama E. (2013). Mechanisms of the immunosuppressive effects of mouse adipose tissue-derived mesenchymal stromal cells on mouse alloreactively stimulated spleen cells. *Experimental and Therapeutic Medicine*.

[133] Le Blanc K., Tammik L., Sundberg B., Haynesworth S. E., Ringdén O. (2003). Mesenchymal stem cells inhibit and stimulate mixed lymphocyte cultures and mitogenic responses independently of the major histocompatibility complex. *Scandinavian Journal of Immunology*.

[134] Schurgers E., Kelchtermans H., Mitera T., Geboes L., Matthys P. (2010). Discrepancy between the *in vitro* and *in vivo* effects of murine mesenchymal stem cells on T-cell proliferation and collagen-induced arthritis. *Arthritis Research & Therapy*.

[135] Wang D., Feng X., Lu L. (2014). A CD8 T cell/indoleamine 2,3-dioxygenase axis is required for mesenchymal stem cell suppression of human systemic lupus erythematosus. *Arthritis and Rheumatology*.

[136] Luz-Crawford P., Kurte M., Bravo-Alegría J. (2013). Mesenchymal stem cells generate a CD4^+^CD25^+^Foxp3^+^ regulatory T cell population during the differentiation process of Th1 and Th17 cells. *Stem Cell Research and Therapy*.

[137] Chinnadurai R., Copland I. B., Patel S. R., Galipeau J. (2014). IDO-independent suppression of T cell effector function by IFN-*γ*-licensed human mesenchymal stromal cells. *The Journal of Immunology*.

[138] Krampera M., Cosmi L., Angeli R. (2006). Role for interferon-*γ* in the immunomodulatory activity of human bone marrow mesenchymal stem cells. *STEM CELLS*.

[139] Haddad R., Saldanha-Araujo F. (2014). Mechanisms of T-cell immunosuppression by mesenchymal stromal cells: what do we know so far?. *BioMed Research International*.

[140] Ghannam S., Pène J., Moquet-Torcy G., Jorgensen C., Yssel H. (2010). Mesenchymal stem cells inhibit human Th17 cell differentiation and function and induce a T regulatory cell phenotype. *Journal of Immunology*.

[141] Duffy M. M., Pindjakova J., Hanley S. A. (2011). Mesenchymal stem cell inhibition of T-helper 17 cell- differentiation is triggered by cell-cell contact and mediated by prostaglandin E2 via the EP4 receptor. *European Journal of Immunology*.

[142] Qu X., Liu X., Cheng K., Yang R., Zhao R. C. H. (2012). Mesenchymal stem cells inhibit Th17 cell differentiation by IL-10 secretion. *Experimental Hematology*.

[143] Benvenuto F., Ferrari S., Gerdoni E. (2007). Human mesenchymal stem cells promote survival of T cells in a quiescent state. *STEM CELLS*.

[144] Cho K.-S., Kim Y.-W., Kang M. J., Park H.-Y., Hong S.-L., Roh H.-J. (2014). Immunomodulatory effect of mesenchymal stem cells on T lymphocyte and cytokine expression in nasal polyps. *Otolaryngology—Head and Neck Surgery*.

[145] Eljaafari A., Tartelin M.-L., Aissaoui H. (2012). Bone marrow-derived and synovium-derived mesenchymal cells promote Th17 cell expansion and activation through caspase 1 activation: contribution to the chronicity of rheumatoid arthritis. *Arthritis and Rheumatism*.

[146] Strober S. (1984). Natural suppressor (NS) cells, neonatal tolerance, and total lymphoid irradiation: exploring obscure relationships. *Annual Review of Immunology*.

[147] Schwadron R. B., Gandour D. M., Strober S. (1985). Cloned natural suppressor cell lines derived from the spleens of neonatal mice. *The Journal of Experimental Medicine*.

[148] Kusmartsev S. A., Li Y., Chen S.-H. (2000). Gr-1^+^ myeloid cells derived from tumor-bearing mice inhibit primary T cell activation induced through CD3/CD28 costimulation. *The Journal of Immunology*.

[149] Youn J.-I., Nagaraj S., Collazo M., Gabrilovich D. I. (2008). Subsets of myeloid-derived suppressor cells in tumor-bearing mice. *The Journal of Immunology*.

[150] Bunt S. K., Clements V. K., Hanson E. M., Sinha P., Ostrand-Rosenberg S. (2009). Inflammation enhances myeloid-derived suppressor cell cross-talk by signaling through Toll-like receptor 4. *Journal of Leukocyte Biology*.

[151] Lesokhin A. M., Hohl T. M., Kitano S. (2012). Monocytic CCR2+ myeloid-derived suppressor cells promote immune escape by limiting activated CD8 T-cell infiltration into the tumor microenvironment. *Cancer Research*.

[152] Iwata Y., Furuichi K., Kitagawa K. (2010). Involvement of CD11b^+^ GR-1low cells in autoimmune disorder in MRL-Fas lpr mouse. *Clinical and Experimental Nephrology*.

[153] Sinha P., Clements V. K., Bunt S. K., Albelda S. M., Ostrand-Rosenberg S. (2007). Cross-talk between myeloid-derived suppressor cells and macrophages subverts tumor immunity toward a type 2 response. *The Journal of Immunology*.

[154] Messmann J. J., Reisser T., Leithäuser F., Lutz M. B., Debatin K.-M., Strauss G. (2015). In vitro-generated MDSCs prevent murine GVHD by inducing type 2 T cells without disabling antitumor cytotoxicity. *Blood*.

[155] Liao J., Wang X., Bi Y. (2014). Dexamethasone potentiates myeloid-derived suppressor cell function in prolonging allograft survival through nitric oxide. *Journal of Leukocyte Biology*.

[156] Gao J., Wu Y., Su Z. (2014). Infiltration of alternatively activated macrophages in cancer tissue is associated with MDSC and Th2 polarization in patients with esophageal cancer. *PLoS ONE*.

[157] Chatterjee S., Das S., Chakraborty P., Manna A., Chatterjee M., Choudhuri S. K. (2013). Myeloid derived suppressor cells (MDSCs) can induce the generation of Th17 response from naïve CD4^+^ T cells. *Immunobiology*.

[158] Bowen J. L., Olson J. K. (2009). Innate immune CD11b^+^Gr-1^+^ cells, suppressor cells, affect the immune response during Theiler's virus-induced demyelinating disease. *Journal of Immunology*.

[159] Zoso A., Mazza E. M. C., Bicciato S. (2014). Human fibrocytic myeloid-derived suppressor cells express IDO and promote tolerance via Treg-cell expansion. *European Journal of Immunology*.

[160] Huang B., Pan P.-Y., Li Q. (2006). Gr-1+CD115+ immature myeloid suppressor cells mediate the development of tumor-induced T regulatory cells and T-cell anergy in tumor-bearing host. *Cancer Research*.

[161] Pan P.-Y., Ma G., Weber K. J. (2010). Immune stimulatory receptor CD40 is required for T-cell suppression and T regulatory cell activation mediated by myeloid-derived suppressor cells in cancer. *Cancer Research*.

[162] Chou H.-S., Hsieh C.-C., Charles R. (2012). Myeloid-derived suppressor cells protect islet transplants by B7-H1 mediated enhancement of T regulatory cells. *Transplantation*.

[163] Jiang X.-X., Zhang Y., Liu B. (2005). Human mesenchymal stem cells inhibit differentiation and function of monocyte-derived dendritic cells. *Blood*.

[164] Nauta A. J., Kruisselbrink A. B., Lurvink E., Willemze R., Fibbe W. E. (2006). Mesenchymal stem cells inhibit generation and function of both CD34^+^-derived and monocyte-derived dendritic cells. *The Journal of Immunology*.

[165] Djouad F., Charbonnier L.-M., Bouffi C. (2007). Mesenchymal stem cells inhibit the differentiation of dendritic cells through an interleukin-6-dependent mechanism. *Stem Cells*.

[166] Spaggiari G. M., Abdelrazik H., Becchetti F., Moretta L. (2009). MSCs inhibit monocyte-derived DC maturation and function by selectively interfering with the generation of immature DCs: central role of MSC-derived prostaglandin E_2_. *Blood*.

[167] Melief S. M., Geutskens S. B., Fibbe W. E., Roelofs H. (2013). Multipotent stromal cells skew monocytes towards an anti-inflammatory interleukin-10-producing phenotype by production of interleukin-6. *Haematologica*.

[168] Liu W.-H., Liu J.-J., Wu J. (2013). Novel mechanism of inhibition of dendritic cells maturation by mesenchymal stem cells via interleukin-10 and the JAK1/STAT3 signaling pathway. *PLoS ONE*.

[169] Liu Y., Yin Z., Zhang R. (2014). MSCs inhibit bone marrow-derived DC maturation and function through the release of TSG-6. *Biochemical and Biophysical Research Communications*.

[170] Zhang Y., Cai W., Huang Q. (2014). Mesenchymal stem cells alleviate bacteria-induced liver injury in mice by inducing regulatory dendritic cells. *Hepatology*.

[171] Liu X., Qu X., Chen Y. (2012). Mesenchymal stem/stromal cells induce the generation of novel IL-10-dependent regulatory dendritic cells by SOCS3 activation. *The Journal of Immunology*.

[172] Rutella S., Bonanno G., Procoli A. (2006). Hepatocyte growth factor favors monocyte differentiation into regulatory interleukin (IL)-10^++^IL-12^low/neg^ accessory cells with dendritic-cell features. *Blood*.

[173] Greenhill C. J., Rose-John S., Lissilaa R. (2011). IL-6 trans-signaling modulates TLR4-dependent inflammatory responses via STAT3. *The Journal of Immunology*.

[174] Zhang H., Hu H., Greeley N. (2014). STAT3 restrains RANK- and TLR4-mediated signalling by suppressing expression of the E2 ubiquitin-conjugating enzyme Ubc13. *Nature Communications*.

[175] Li Y.-P., Paczesny S., Lauret E. (2008). Human mesenchymal stem cells license adult CD34^+^ hemopoietic progenitor cells to differentiate into regulatory dendritic cells through activation of the notch pathway. *Journal of Immunology*.

[176] Aldinucci A., Rizzetto L., Pieri L. (2010). Inhibition of immune synapse by altered dendritic cell actin distribution: a new pathway of mesenchymal stem cell immune regulation. *The Journal of Immunology*.

[177] Chiesa S., Morbelli S., Morando S. (2011). Mesenchymal stem cells impair in vivo T-cell priming by dendritic cells. *Proceedings of the National Academy of Sciences of the United States of America*.

[178] Biswas S. K., Mantovani A. (2010). Macrophage plasticity and interaction with lymphocyte subsets: cancer as a paradigm. *Nature Immunology*.

[179] Gao S., Mao F., Zhang B. (2014). Mouse bone marrow-derived mesenchymal stem cells induce macrophage M2 polarization through the nuclear factor-*κ*B and signal transducer and activator of transcription 3 pathways. *Experimental Biology and Medicine*.

[180] Kim J., Hematti P. (2009). Mesenchymal stem cell-educated macrophages: a novel type of alternatively activated macrophages. *Experimental Hematology*.

[181] Németh K., Leelahavanichkul A., Yuen P. S. T. (2009). Bone marrow stromal cells attenuate sepsis via prostaglandin E_2_-dependent reprogramming of host macrophages to increase their interleukin-10 production. *Nature Medicine*.

[182] Choi H., Lee R. H., Bazhanov N., Oh J. Y., Prockop D. J. (2011). Anti-inflammatory protein TSG-6 secreted by activated MSCs attenuates zymosan-induced mouse peritonitis by decreasing TLR2/NF-*κ*B signaling in resident macrophages. *Blood*.

[183] François M., Romieu-Mourez R., Li M., Galipeau J. (2012). Human MSC suppression correlates with cytokine induction of indoleamine 2,3-dioxygenase and bystander M2 macrophage differentiation. *Molecular Therapy*.

[184] Zhang Q.-Z., Su W.-R., Shi S.-H. (2010). Human gingiva-derived mesenchymal stem cells elicit polarization of M2 macrophages and enhance cutaneous wound healing. *Stem Cells*.

[185] Maron-Gutierrez T., Silva J. D., Asensi K. D. (2013). Effects of mesenchymal stem cell therapy on the time course of pulmonary remodeling depend on the etiology of lung injury in mice. *Critical Care Medicine*.

[186] Ionescu L., Byrne R. N., van Haaften T. (2012). Stem cell conditioned medium improves acute lung injury in mice: in vivo evidence for stem cell paracrine action. *The American Journal of Physiology—Lung Cellular and Molecular Physiology*.

[187] Poschke I., Mao Y., Adamson L., Salazar-Onfray F., Masucci G., Kiessling R. (2012). Myeloid-derived suppressor cells impair the quality of dendritic cell vaccines. *Cancer Immunology, Immunotherapy*.

[188] Hu C.-E., Gan J., Zhang R.-D., Cheng Y.-R., Huang G.-J. (2011). Up-regulated myeloid-derived suppressor cell contributes to hepatocellular carcinoma development by impairing dendritic cell function. *Scandinavian Journal of Gastroenterology*.

[189] Corcione A., Benvenuto F., Ferretti E. (2006). Human mesenchymal stem cells modulate B-cell functions. *Blood*.

[190] Franquesa M., Mensah F. K., Huizinga R. (2015). Human adipose tissue-derived mesenchymal stem cells abrogate plasmablast formation and induce regulatory B cells independently of T helper cells. *Stem Cells*.

[191] Rosado M. M., Bernardo M. E., Scarsella M. (2015). Inhibition of B-cell proliferation and antibody production by mesenchymal stromal cells is mediated by T cells. *Stem Cells and Development*.

[192] Traggiai E., Volpi S., Schena F. (2008). Bone marrow-derived mesenchymal stem cells induce both polyclonal expansion and differentiation of B cells isolated from healthy donors and systemic lupus erythematosus patients. *STEM CELLS*.

[193] Rasmusson I., Le Blanc K., Sundberg B., Ringdén O. (2007). Mesenchymal stem cells stimulate antibody secretion in human B cells. *Scandinavian Journal of Immunology*.

[194] Qin Y., Zhou Z., Zhang F. (2015). Induction of regulatory B-cells by mesenchymal stem cells is affected by SDF-1*α*-CXCR7. *Cellular Physiology and Biochemistry*.

[195] Peng Y., Chen X., Liu Q. (2015). Mesenchymal stromal cells infusions improve refractory chronic graft versus host disease through an increase of CD5^+^ regulatory B cells producing interleukin 10. *Leukemia*.

[196] Asari S., Itakura S., Ferreri K. (2009). Mesenchymal stem cells suppress B-cell terminal differentiation. *Experimental Hematology*.

[197] O'Connor M. A., Fu W. W., Green K. A., Green W. R. (2015). Subpopulations of M-MDSCs from mice infected by an immunodeficiency-causing retrovirus and their differential suppression of T- vs B-cell responses. *Virology*.

[198] Green K. A., Wang L., Noelle R. J., Greena W. R. (2015). Selective involvement of the checkpoint regulator VISTA in suppression of B-cell, but not T-cell, responsiveness by monocytic myeloid-derived suppressor cells from mice infected with an immunodeficiency-causing retrovirus. *Journal of Virology*.

[199] Kennedy D. E., Knight K. L. (2015). Inhibition of B lymphopoiesis by adipocytes and IL-1-producing myeloid-derived suppressor cells. *Journal of Immunology*.

[200] Crook K. R., Jin M., Weeks M. F. (2015). Myeloid-derived suppressor cells regulate T cell and B cell responses during autoimmune disease. *Journal of Leukocyte Biology*.

[201] Sotiropoulou P. A., Perez S. A., Gritzapis A. D., Baxevanis C. N., Papamichail M. (2006). Interactions between human mesenchymal stem cells and natural killer cells. *Stem Cells*.

[202] Chatterjee D., Tufa D. M., Baehre H., Hass R., Schmidt R. E., Jacobs R. (2014). Natural killer cells acquire CD73 expression upon exposure to mesenchymal stem cells. *Blood*.

[203] Goh C. C., Roggerson K. M., Lee H. C., Golden-Mason L., Rosen H. R., Hahn Y. S. (2016). Hepatitis C virus-induced myeloid-derived suppressor cells suppress NK cell IFN-*γ* production by altering cellular metabolism via arginase-1. *The Journal of Immunology*.

[204] Wong J. L., Obermajer N., Odunsi K., Edwards R. P., Kalinski P. (2016). Synergistic COX2 induction by IFN*γ* and TNF*α* self-limits type-1 immunity in the human tumor microenvironment. *Cancer Immunology Research*.

[205] Draghiciu O., Lubbers J., Nijman H. W., Daemen T. (2015). Myeloid derived suppressor cells—an overview of combat strategies to increase immunotherapy efficacy. *OncoImmunology*.

[206] Zhu J., Huang X., Yang Y. (2012). Myeloid-derived suppressor cells regulate natural killer cell response to adenovirus-mediated gene transfer. *The Journal of Virology*.

[207] Raffaghello L., Bianchi G., Bertolotto M. (2008). Human mesenchymal stem cells inhibit neutrophil apoptosis: a model for neutrophil preservation in the bone marrow niche. *Stem Cells*.

[208] Cassatella M. A., Mosna F., Micheletti A. (2011). Toll-like receptor-3-activated human mesenchymal stromal cells significantly prolong the survival and function of neutrophils. *STEM CELLS*.

[209] Brandau S., Jakob M., Hemeda H. (2010). Tissue-resident mesenchymal stem cells attract peripheral blood neutrophils and enhance their inflammatory activity in response to microbial challenge. *Journal of Leukocyte Biology*.

[210] Hall S. R. R., Tsoyi K., Ith B. (2013). Mesenchymal stromal cells improve survival during sepsis in the absence of heme oxygenase-1: the importance of neutrophils. *Stem Cells*.

[211] Hsu S.-C., Wang L.-T., Yao C.-L. (2013). Mesenchymal stem cells promote neutrophil activation by inducing IL-17 production in CD4^+^ CD45RO^+^ T cells. *Immunobiology*.

[212] Poe S. L., Arora M., Oriss T. B. (2013). STAT1-regulated lung MDSC-like cells produce IL-10 and efferocytose apoptotic neutrophils with relevance in resolution of bacterial pneumonia. *Mucosal Immunology*.

[213] Ray A., Chakraborty K., Ray P. (2013). Immunosuppressive MDSCS induced by TLR signaling during infection and role in resolution of inflammation. *Frontiers in Cellular and Infection Microbiology*.

[214] Chen H. W., Chen H.-Y., Wang L.-T. (2013). Mesenchymal stem cells tune the development of monocyte-derived dendritic cells toward a myeloid-derived suppressive phenotype through growth-regulated oncogene chemokines. *The Journal of Immunology*.

[215] Waterman R. S., Tomchuck S. L., Henkle S. L., Betancourt A. M. (2010). A new mesenchymal stem cell (MSC) paradigm: polarization into a pro-inflammatory MSC1 or an immunosuppressive MSC2 phenotype. *PLoS ONE*.

[216] Okamoto R., Watanabe M. (2016). Investigating cell therapy for inflammatory bowel disease. *Expert Opinion on Biological Therapy*.

[217] Cras A., Farge D., Carmoi T., Lataillade J.-J., Wang D. D., Sun L. (2015). Update on mesenchymal stem cell-based therapy in lupus and scleroderma. *Arthritis Research & Therapy*.

[218] Dulamea A. (2015). Mesenchymal stem cells in multiple sclerosis—translation to clinical trials. *Journal of Medicine and Life*.

[219] Llufriu S., Sepúlveda M., Blanco Y. (2014). Randomized placebo-controlled phase II trial of autologous mesenchymal stem cells in multiple sclerosis. *PLoS ONE*.

[220] Rizk M., Monaghan M., Shorr R., Kekre N., Bredeson C. N., Allan D. S. (2016). Heterogeneity in studies of mesenchymal stromal cells to treat or prevent graft-versus-host disease: a scoping review of the evidence. *Biology of Blood and Marrow Transplantation*.

[221] Chen C., Hou J. (2016). Mesenchymal stem cell-based therapy in kidney transplantation. *Stem Cell Research & Therapy*.

[222] Cerqueira M. T., Pirraco R. P., Marques A. P. (2016). Stem cells in skin wound healing: are we there yet?. *Advances in Wound Care*.

[223] Ezquer F. (2016). Multipotent mesenchymal stromal cells: A promising strategy to manage alcoholic liver disease. *World Journal of Gastroenterology*.

[224] Wakitani S., Okabe T., Horibe S. (2011). Safety of autologous bone marrow-derived mesenchymal stem cell transplantation for cartilage repair in 41 patients with 45 joints followed for up to 11 years and 5 months. *Journal of Tissue Engineering and Regenerative Medicine*.

[225] Hare J. M., Traverse J. H., Henry T. D. (2009). A randomized, double-blind, placebo-controlled, dose-escalation study of intravenous adult human mesenchymal stem cells (prochymal) after acute myocardial infarction. *Journal of the American College of Cardiology*.

[226] Savukinas U. B., Enes S. R., Sjöland A. A., Westergren-Thorsson G. (2016). The bystander effect: MSC-mediated lung repair. *Stem Cells*.

[227] Otero-Viñas M., Falanga V. (2016). Mesenchymal stem cells in chronic wounds: the spectrum from basic to advanced therapy. *Advances in Wound Care*.

[228] Di Mitri D., Toso A., Alimonti A. (2015). Molecular pathways: targeting tumor-infiltrating myeloid-derived suppressor cells for cancer therapy. *Clinical Cancer Research*.

[229] Markowitz J., Wesolowski R., Papenfuss T., Brooks T. R., Carson W. E. (2013). Myeloid-derived suppressor cells in breast cancer. *Breast Cancer Research and Treatment*.

[230] Stoermer K. A., Burrack A., Oko L. (2012). Genetic ablation of arginase 1 in macrophages and neutrophils enhances clearance of an arthritogenic alphavirus. *Journal of Immunology*.

[231] Arora M., Poe S. L., Oriss T. B. (2010). TLR4/MyD88-induced CD11b^+^ Gr-1 int F4/80^+^ non-migratory myeloid cells suppress Th2 effector function in the lung. *Mucosal Immunology*.

